# Predicting future onset of depression among middle-aged adults with no psychiatric history

**DOI:** 10.1192/bjo.2023.62

**Published:** 2023-05-23

**Authors:** Yonatan Bilu, Nir Kalkstein, Eva Gilboa-Schechtman, Pinchas Akiva, Gil Zalsman, Liat Itzhaky, Dana Atzil-Slonim

**Affiliations:** KI Research Institute, Kfar Malal, Israel; Department of Psychology, Bar-Ilan University, Israel; Psychiatry School of Continuing Medical Education, Tel-Aviv University, Israel; Department of Psychiatry, Columbia University Medical Center, New York, USA

**Keywords:** Depressive disorders, information technologies, machine learning, predictive model, UK Biobank

## Abstract

**Background:**

Depression is a major cause of disability worldwide. Recent data suggest that, in industrialised countries, the prevalence of depression peaks in middle age. Identifying factors predictive of future depressive episodes is crucial for developing prevention strategies for this age group.

**Aims:**

We aimed to identify future depression in middle-aged adults with no previous psychiatric history.

**Method:**

To predict a diagnosis of depression 1 year or more following a comprehensive baseline assessment, we used a data-driven, machine-learning methodology. Our data-set was the UK Biobank of middle-aged participants (*N* = 245 036) with no psychiatric history.

**Results:**

Overall, 2.18% of the study population developed a depressive episode at least 1 year following baseline. Basing predictions on a single mental health questionnaire led to an area under the curve of the receiver operating characteristic of 0.66, and a predictive model leveraging the combined results of 100 UK Biobank questionnaires and measurements improved this to 0.79. Our findings were robust to demographic variations (place of birth, gender) and variations in methods of depression assessment. Thus, machine-learning-based models best predict diagnoses of depression when allowing the inclusion of multiple features.

**Conclusions:**

Machine-learning approaches show potential for being beneficial for the identification of clinically relevant predictors of depression. Specifically, we can identify, with moderate success, people with no recorded psychiatric history as at risk for depression by using a relatively small number of features. More work is required to improve these models and evaluate their cost-effectiveness before integrating them into the clinical workflow.

## Background

Depression is a major cause of disability,^1^ and its personal, societal and economic impacts make it crucial to expand knowledge concerning mitigating strategies. Identifying the risk factors that are most predictive of future depressive symptoms may help improve depression prevention strategies. The prevalence of depression differs by age,^2^ and recent studies show that in high-income countries, the prevalence of depression is highest among middle-aged individuals.^3,4^ The literature on risk factors for depression suggests that although many risk factors are common across the lifespan,^5^ there are factors that are age specific.^6–9^ Previous studies have tended to examine risk factors among other age groups, such as adolescents,^7^ young adults^8^ and older adults,^9^ but there has been little consideration of risk factors among middle-aged adults. Elucidating the risk factors among middle-aged adults – the high-risk age for depression – may allow for the development of age-specific interventions. Many risk factors have been identified as contributing to the development of depression.^10^ Despite the growing acknowledgment that depression is a complex and multifactorial disorder,^11^ recent literature reviews of depression risk factors point to a common limitation of existing studies: most of them focused on validating sets of hypothesised risk factors associated with depression, and examined each risk factor separately.^10,12^ It has been suggested that to obtain a broader view of modifiable factors that can be used to identify and prevent depressive episodes, it is necessary to examine a wide range of factors in unison.^10,12^ The paucity of studies examining multiple risk factors may be because of the need for large sample sizes for such an analysis,^10^ and methodologies appropriate for multidimensional data.^12^ Most previous studies were based on small sample sizes and used classical inferential analytic approach, resulting in low predictive strength of identified risk factors.^12^ Prominent researchers have recently suggested that the understanding of risk factors for depression could be substantially enriched by the use of machine-learning techniques that can handle rich and complex data.^10,12,13^ The availability of large data-sets such as the UK Biobank,^14^ and the development of machine-learning techniques that can access and analyse such data-sets, allows for comprehensive and well-powered examinations.

Several recent studies have used the UK Biobank to identify risk factors for depression,^15–17^ demonstrating the usefulness of applying machine-learning techniques to a large and rich data-set. However, these studies predefined theoretically derived risk factors. A data-driven approach may enable the discovery of the factors that are most predictive of depression, along with their relative contributions. Only a single study has used machine-learning models to predict future depression.^18^ However, the UK Biobank allows for analysis on a much greater scale.

## Aims and contribution

The current study aims to use a data-driven approach to analyse the UK Biobank data-set and identify the risk factors that are most predictive of future depression among middle-aged adults. We focus on people with no history of mental health illnesses, since we expect that they are more likely to benefit from this than those who have already interacted with the mental health system. Moreover, since mental health history is a good predictor for future depression,^10^ the excluded cases are those for which the prediction task is easier. Specifically, we are interested in early identification (at least 1 year before diagnosis) of people at risk of depression, as this time frame may mitigate, or even prevent, the onset of depression; for example, by raising their awareness to the mental health services that are available to them. Importantly, this should be done with caution, so as not to become a ‘self-fulfilling prophecy’. Additionally, we aim to (a) evaluate the predictive power of features derived from the UK Biobank beyond those that only concern mental health, (b) select a small set of features that are most predictive of future depression and (c) evaluate the robustness of the resultant predictive models.

## Method

### Sample

The UK Biobank^13^ contains medical data for 502 504 individuals; all participants were aged 40–70 years at recruitment (which occurred in 2006–2010). The data include a large array of questionnaire results, laboratory test results, demographic information and ICD-10 medical diagnoses, which are aggregated from primary care and secondary care records and self-reports.

Middle age is usually defined as 40 to 60–65 years.^19,20^ Accordingly, we included all participants who were aged 40–60 at recruitment and who had no diagnoses of mental health issues (any diagnosis for which the ICD-10 code begins with ‘F’) before the baseline assessment. We did not censor the cohort based on the age at which depression was diagnosed, as this would bias the data by allowing younger participants a longer time window for diagnosis. For most participants diagnosed with depression (81.75%), diagnosis was within 7 years of recruitment, with the longest period being 11.6 years after baseline. Almost all of those diagnosed with depression (97.3%) were diagnosed by age 65 years.

The baseline UK Biobank data pertain to all participants; however, additional data were collected at later dates for participant subsets. To obtain a large cohort for our analysis, we included data that were available for a majority of participants and that afforded classification (for details, see Supplementary File 1 available at https://doi.org/10.1192/bjo.2023.62). As some data were country specific (e.g. income score was listed separately for England, Wales and Scotland), we merged these data when possible. Overall, we extracted 481 data values for each participant.

The final cohort comprised 245 036 participants, of which 5345 were diagnosed with depression at least 1 year after baseline (‘positive examples’; 2.18%). Among the 133 806 men and 111 230 women included in this cohort, 3373 (2.52%) men and 1972 (1.77%) women were positive examples.

### Future depression

For each participant, the main UK Biobank data table includes the first date of a diagnosis of an ICD-10-registered illness. We defined a diagnosis of depression as a data entry comprising the ICD-10 code ‘F32’. This approach was advantageous because the relevant data table is provided for all participants, and because the diagnosis was not influenced by the present study goals.

We tested the robustness of this approach by examining how well one can use a model trained on ICD-10 data to predict depression as defined by the Patient Health Questionnaire-9 (PHQ-9;^21^ a score of ≥15). The same definitions for depression have previously been used to classify UK Biobank participants.^22^

### Prediction and evaluation

#### Feature extraction

To model the participants and predict future onset of depression, we transformed the extracted UK Biobank data into binary feature vectors. For example, one UK Biobank question concerned types of physical activity performed in the past 4 weeks. Possible answers were walking for pleasure, other activity, strenuous sports, light DIY and heavy DIY. This question creates six potential features: one for each of the possible answers, and an additional one for no response. Participants who indicated that they walked for pleasure (and nothing else) would be awarded a value of ‘1’ for the first feature, and ‘0’ for the others (see Supplementary File 1 for details). Overall, this transformation yielded a binary feature matrix with 245 036 rows and 2851 columns.

#### Prediction

Data were partitioned into six subsets based on participants’ countries of birth (see Table 1). We set the subset of those born in England as our development set, and the others as test sets, which could be considered subjects for external validations or subgroup analysis. The main analysis was done on the development set, and the remainder were used for validation.

**Table 1:** Number of participants in each external validation set, defined by country of birth, and the number in each set who were diagnosed with depression at least 1 year after the baseline assessment

Country	Number of participants	Number of positive examples	Per cent
England	184 669	3964	2.15%
Wales	10 939	364	3.33%
Scotland	20 622	488	2.37%
Northern Ireland	1480	31	2.09%
Republic of Ireland	1878	44	2.34%
Elsewhere	24 437	425	1.74%

As a first step, for each feature in the UK Biobank we trained a classifier that aims to predict future depression based solely on this feature, and evaluated its predictive power. Then we defined subsets of features and built prediction models based on them.

Namely, different subsets of features were used to train seven different models: *Model_Best-500_,* and *Model_Best-100_* were trained on the top 500 and 100 features, respectively; *Model_NMH-500_* and *Model_NMH-100_* were similarly trained on the top features, excluding mental health features; *Model_MH_* was trained solely on mental health features; *Model_LS_* was trained only on features reflecting lifestyle and environment, with *Model_LS-100_* being trained on the top 100 features of this type. See Supplementary File 1 for more detail.

#### Evaluation metrics

Models were evaluated based on how well they identified participants who were diagnosed with depression at least 1 year after baseline. This evaluation was conducted through a ten-fold cross-validation of the development set. Specifically, data were randomly partitioned into two subsets, one containing 70% of the data and the other containing 30% of the data. Predictive models were constructed using the larger set, and then evaluated on the smaller set. This was repeated ten times, to obtain robust results.

Three evaluation metrics were considered. The first two focus on the 5% of participants who are predicted to be most at risk. One metric (precision at 5%, denoted Pr-5%) computes the fraction of participants that indeed have a future diagnosis of depression. The other (recall at 5%, denoted Re-5%) computes what fraction of all participants with a future diagnosis of depression are included in this top 5%.

The third evaluation metric, area under the curve of the receiver operating characteristic (AUC), looks at the graph of true positive rate versus false positive rate, and computes the area under that graph. See Supplementary File 1 for exact definitions and motivations for using these metrics. For our purposes, the important feature of the AUC is that it reflects the quality of the entire set of predictions, rather than just the top 5%.

#### Evaluating the robustness of the predictive models

##### Demographic variables (place of birth and gender)

To evaluate the robustness of our predictive models, we first constructed models based on the development set, and evaluated them on each of the left-out sets (those comprising participants born outside of England). The number of participants in each such set is listed in Table 1. The incidence of ‘future depression’ varied among these cohorts, being highest among those born in Wales (3.33%) and lowest among those born outside of the UK and Ireland (1.74%). Note that this does not necessarily imply that this is the relative incidence of depression among these groups because we were considering ‘future depression’ and specifically excluded all participants with a history of mental health issues; also, there may be a bias among these groups regarding diagnosis of depression. As with the cross-validation experiments, we examined several models, defined by the type of UK Biobank data entries they were trained on.

Next, we examined the model's sensitivity to the participants’ gender. We partitioned the data into men and women, and trained the models on one group and then evaluated them on the other; this was performed in both directions.

##### Definition of depression (ICD-10 versus PHQ-9)

Finally, we examined the sensitivity of the prediction models to the method used to define depression. Specifically, we split the participants into those who completed the questionnaire and those who did not. The first group comprised 83 654 participants, among whom 1.7% scored ≥15 on the PHQ-9. This set was used for validation, with the goal being to identify participants with similarly high scores. The second group comprised 161 382 participants, among which 2.4% had an ICD-10 diagnosis of depression. This set was then used to train the models, as in the other experiments.

### Ethical approval

UK Biobank has received ethical approval from the UK National Health Service's National Research Ethics Service (reference 11/ NW/0382), and this work is part of project number 83 122. As the research is based on the UK Biobank data, informed consent and the Declaration of Helsinki were not relevant/necessary.

## Results

### Cross-validation

We first sought features that are independently predictive of future diagnosis of depression. Table 2 lists the UK Biobank columns that independently produced the most successful classifiers. As expected, entries associated with questions regarding current and past mental health were best for predicting future depression. Additionally, measures of general health, physical activity, smoking, financial circumstances and body mass all represented relatively good predictors.

**Table 2:** Evaluation statistics for models derived from single UK Biobank columns, averaged over ten folds of cross-validation

UK Biobank category	UK Biobank column	AUC	Pr-5%	Re-5%
Diet	Major dietary changes in the past 5 years	0.57	0.043	0.10
Employment	Current employment status	0.56	0.061	0.14
General health	Falls in the past year	0.55	0.052	0.12
General health	Overall health rating	0.64	0.066	0.16
Household	Average total household income before tax	0.62	0.043	0.10
Household	Number of vehicles in household	0.57	0.043	0.10
Household	Own or rent accommodation lived in	0.57	0.058	0.14
Impedance measures	Body mass index (BMI)	0.58	0.042	0.10
Medical conditions	Number of self-reported non-cancer illnesses	0.61	0.041	0.10
Medications	Number of treatments/medications taken	0.64	0.054	0.13
Operations	Number of operations, self-reported	0.59	0.042	0.10
Pain	Pain type(s) experienced in the past month	0.62	0.043	0.10
Physical activity	Time spent watching television (TV)	0.57	0.041	0.10
Physical activity	Types of physical activity in the past 4 weeks	0.56	0.047	0.11
Physical activity	Types of transport used (excluding work)	0.53	0.043	0.10
Physical activity	Usual walking pace	0.58	0.058	0.14
Sleep	Daytime dozing/sleeping (narcolepsy)	0.55	0.040	0.10
Sleep	Getting up in morning	0.58	0.050	0.12
Smoking	Current tobacco smoking	0.53	0.043	0.10
Smoking	Exposure to tobacco smoke outside of the home	0.56	0.041	0.10
Smoking	Past tobacco smoking	0.55	0.040	0.10
Mental health	Frequency of depressed mood^a^	0.66	0.069	0.16
Mental health	Frequency of tenseness/restlessness^a^	0.64	0.063	0.15
Mental health	Frequency of tiredness/lethargy^a^	0.64	0.060	0.14
Mental health	Frequency of unenthusiasm/disinterest^a^	0.63	0.059	0.14
Mental health	Loneliness, isolation	0.61	0.048	0.11
Mental health	Mood swings	0.64	0.040	0.10
Mental health	Neuroticism score	0.68	0.065	0.15
Mental health	Seen a psychiatrist^b^	0.57	0.062	0.15
Mental health	Seen doctor (general practitioner)^b^	0.70	0.055	0.13
Mental health	Tense/‘highly strung’	0.59	0.045	0.11

Columns with a Pr-5% value >0.04 are shown. Several ‘impedance measures’ attain values similar to those listed for body mass index, and are not shown. See the UK Biobank site (https://biobank.ndph.ox.ac.uk/showcase/search.cgi) for more details on these entries. AUC, area under the curve of the receiver operating characteristic; Pr-5%, precision at 5%; Re-5%, recall at 5%.

a Past 2 weeks.

b For nervousness, anxiety, tension or depression.

Our main research question concerned the predictive power of multi-feature classifiers. Table 3 details the evaluation of the main model, which had access to all UK Biobank columns, as well as models restricted to specific features. This shows that, although the predictive power of features associated with mental health was high, they were not independently sufficient to match the quality of the full model. Furthermore, omitting these features did not greatly reduce the predictive power. Limiting the model to features describing lifestyle and environment also limited the success of the prediction model, reducing Pr-5% from 12% to 8%.

**Table 3:** Evaluation statistics for the models derived from all features and from mental health and lifestyle features

Model	Number of features	Number of columns	AUC	Pr-5%	Re-5%
*Model_Best-500_*	500	288.10	0.79	0.12	0.27
*Model_Best-100_*	100	68.20	0.79	0.12	0.27
*Model_MH_*	113	27.00	0.78	0.10	0.25
*Model_NMH-500_*	500	279.50	0.73	0.10	0.23
*Model_NMH-100_*	100	79.80	0.72	0.09	0.22
*Model_LS_*	536	88.00	0.68	0.08	0.17
*Model_LS-100_*	100	57.30	0.68	0.08	0.18

The third column in the table represents the number of UK Biobank columns from which the features were derived, averaged over the ten folds of cross-validation. AUC, area under the curve of the receiver operating characteristic; Pr-5%, precision at 5%; Re-5%, recall at 5%.

For the full model, for those predicted to be most at risk, precision was over six times that expected at random: 2.2% of the participants in the entire data-set developed depression, whereas among the 5% predicted to be most at risk, 12% developed depression. When examining only 5% of the population, the model identified over a quarter of those who developed depression. It is interesting to compare these numbers with those from Table 2. Evidently, combining multiple features provides more precise predictions.

Next, we investigated whether a concise set of features is sufficient for high-quality predictions. Supplementary Fig. 1 depicts the evaluation metrics as a function of the number of features selected. This shows that adding more features tends to improve classification, but only moderately, and this generally plateaus after 100 features. The highest AUC of 0.8 was attained by *Model_Best−_*_300_, but ten features obtained an AUC of 0.76 (averaged over ten folds).

Limiting the analysis to a single gender yielded similar results, although, rather than plateauing, metrics seemed to decline. This may be attributed to overfitting over these smaller training sets.

### Robustness of the predictive models

#### Sensitivity to demographic variables

Table 4 details the evaluation of models trained on the development set (data from participants born in England) and applied to each of the test sets. The results were similar to those obtained in the cross-validation. Note, for a random baseline, the Pr-5% is expected to be the fraction of positive examples, whereas the Re-5% is expected to be 5%. Hence, higher precision was expected for the Wales-born cohort, and lower precision for the ‘born elsewhere’ cohort. The surprisingly good predictions for the Irish-born cohorts should be considered cautiously, as these cohorts were small, and contained relatively few positive examples.

**Table 4:** Robustness of evaluation statistics to demographic characteristics and depression assessment method

Model evaluation set	Best, AUC; Pr-5%; Re-5%	Mental health, AUC; Pr-5%; Re-5%	Excluding mental health, AUC; Pr-5%; Re-5%	Lifestyle and environment, AUC; Pr-5%; Re-5%
Country of birth				
Wales	0.77; 0.15; 0.22	0.74; 0.12; 0.19	0.73; 0.12; 0.18	0.67; 0.07; 0.11
Scotland	0.73; 0.07; 0.15	0.72; 0.08; 0.18	0.66; 0.06; 0.14	0.64 0.06; 0.12
Northern Ireland	0.77; 0.09; 0.23	0.78; 0.09; 0.23	0.71; 0.12; 0.29	0.78 0.12; 0.29
Republic of Ireland	0.78; 0.13; 0.27	0.76; 0.11; 0.23	0.72; 0.14; 0.30	0.71; 0.09; 0.18
Elsewhere	0.79; 0.10; 0.28	0.77; 0.08; 0.23	0.72; 0.08; 0.23	0.66; 0.06; 0.16
Gender				
Female	0.78; 0.10; 0.28	0.77; 0.09; 0.26	0.71; 0.07; 0.21	0.67; 0.06; 0.18
Male	0.77; 0.12; 0.24	0.76; 0.10; 0.20	0.68; 0.09; 0.18	0.65; 0.07; 0.15
Assessment				
PHQ-9	0.82; 0.12; 0.35	0.81; 0.11; 0.34	0.77; 0.10; 0.31	0.75; 0.09; 0.27

Country of birth section: models trained over the development set and evaluated over the five omitted sets, defined by country of birth. Gender section: models trained over data for one gender and evaluated over data for the other. Assessment section: models trained over data (from the ICD-10) for participants who did not complete the PHQ-9 questionnaire and evaluated over data for those that did, taking ‘severe’ and ‘moderately severe’ PHQ-9 assessments as indications of depression. ‘Best’ models are Best-500 for ‘Country of birth’ and ‘Assessment’, and Best-100 for ‘Gender’. AUC, area under the curve of the receiver operating characteristic; Pr-5%, precision at 5%; Re-5%, recall at 5%; PHQ-9, Patient Health Questionnaire-9.

Importantly, results for the ‘born elsewhere’ set were similar to those obtained for the other sets. This further suggests that the models developed were robust to changes in country of birth, and that they can be deployed elsewhere without requiring further calibration.

Using the same methodology, we trained prediction models on the data for participants listed as male, and evaluated them on the data of those listed as female, and *vice versa*. Table 4 shows that the results were somewhat poorer than when training on the entire population, but overall similar. Interestingly, although future depression was less common among women in our data, and therefore more difficult to detect, especially when learning from examples based on male-sourced data, higher measures were attained in the setting where female-based data were used for evaluation.

#### Sensitivity to definition of depression

Finally, we trained models on data for participants who did not complete the UK Biobank mental health questionnaire. We then tested it on those who did, but set the ground truth labels according to whether their PHQ-9 score was at least 15, rather than ICD-10 diagnoses. Table 4 shows that the predictions were at least comparable to those reported in the previous two sections. This suggests that the predictive models captured a relatively robust definition of depression and were not very sensitive to the way in which it was defined.

## Discussion

Although risk factors for depression onset have been extensively investigated, most studies attempted to validate a limited set of theoretically derived risk factors, tended to examine each risk factor separately across relatively small samples and focused on young or older adults. In contrast, we used a data-driven, machine-learning technique to explore, among a large and rich data-set, the power of multiple factors to predict future depression among middle-aged individuals with no psychiatric history.

The features we found to be the best independent predictors for future depression among middle-aged adults with no psychiatric history are consistent with those identified in other works.^5^ Measures of diet, employment and financial circumstances, general health, physical activity, sleeping habits and smoking represented relatively good predictors of future depression. Associations between some of these factors and depression have also been found in studies of other age groups that examined each of these features separately.^23,24^ Our findings highlight the robustness of the associations between these features and depression among middle-aged adults, and also show the importance of combining multiple features, which produces the highest accuracy. For example, although current depressed mood is a good predictor for future depression (The ‘Frequency of depressed mood in the past 2 weeks’ question achieves a Pr-5% of 0.069), it is not as good as the full model (Pr-5% of 0.12). Indeed, features associated with questions regarding current and past mental health were best for predicting future depression; however, non-mental health features can independently capture most relevant information obtained by mental health features and attain similar precision. This supports the importance of performing a comprehensive assessment of risk factors for depression, to recognise those at high risk of its onset.

Although we excluded participants with mental health diagnoses before baseline or within the subsequent year, questions regarding current mental health were the most useful for predicting future depression. One possible explanation for this is that, although the diagnoses appeared a year or more after baseline, some participants may have been experiencing early stages of (unrecorded) depression during the assessment.

As highlighted previously,^5^ the identification of risk factors, some of which might be modifiable, may be useful for designing more effective preventative strategies for depression. Although, based on our findings, inferences can be made regarding the relationship between risk factors and depression, no conclusions concerning causality can be drawn. It is possible that the causality is the inverse; that is, rather than lifestyle choices leading to depression, early stages of depression may be reflected in one's lifestyle, or lifestyle may be a marker of other underlying causes of depression. A more careful causal analysis or prospective experiments are required to examine whether changes in the multiple features identified in our data-driven study reduce the risk of depression.

As we aimed to identify a relatively small subset of people who were aged 40–60 years, had no recorded history of mental health issues and had a much higher likelihood of future diagnosis of depression than the rest of the population, we focused on precision and recall measures. Our results showed that, among those predicted to be most at risk, precision was over six times that expected at random. Similarly, considering only 5% of the population, the model identified over a quarter of those who developed depression. Evidently, the combination of multiple features leads to more precise predictions. Concurrently, our results show that, although adding more features to the model tended to improve the model's classification (AUC = 0.8 for a model with 300 features), almost equally good results were obtained using far fewer features (AUC = 0.76 for a model with ten features). This finding may have clinical implications for the development of short questionnaires for identifying individuals at risk of developing depression. See the supplementary material for an explicit model for predicting such future depression, based on ten UK Biobank questions.

To evaluate the robustness of our predictive models, we conducted two sets of analyses. The first analysis examined whether the models developed using data for England-born participants can be applied to participants born elsewhere. Our subgroup analysis indicated that, at least when applied to people currently living in the UK, the model was robust to changes in this demographic. Even when evaluating the model on participants born outside of England, the evaluation metrics attained similar values to those in the cross-validation experiment.

The second analysis concerned the effect of gender. The female/male depression prevalence ratio in our sample differed somewhat from that documented in previous epidemiological studies concerning the general population, in which depression diagnosis has typically been reported as being twice as common in women than men.^25^ However, recent studies show that, among the middle-aged population, there are no gender differences concerning the prevalence of depression, and that there may even be a higher prevalence of severe depression among middle-aged men than women.^3^ Indeed, our results suggest that predictions are of similar quality when performed separately for men and women. Additionally, a model trained to predict depression for one gender had generally good performance when applied to the opposite gender. It is worth noting, however, that in both scenarios better measures were attained when trying to predict depression among women.

The final analyses examined whether the models trained to predict depression based on ICD-10 diagnosis would obtain similar results when other accepted definitions of depression are used (in this case, the PHQ-9).^18^ The results attained similar values, further supporting the robustness of our models and suggesting that the models do not depend on the exact depression assessment method.

### Limitations and future directions

This study utilised the relatively readily available UK Biobank data. The features that the models selected to use were mostly sourced from questionnaires. Although depression risk has a hereditary component,^26^ this study did not incorporate features based on genetic data. Similarly, we did not include data that are not available for all participants, so as not to reduce the cohort's size. Further studies incorporating biological, genetic, neuroimaging, precipitating life events and partially available features may increase our understanding of risk factors predictive of future depression in this and other age groups.

Although our results demonstrated robustness across different places of birth, it is possible that variations in other demographic characteristics affect the model's accuracy. Indeed, although results for men and women were approximately similar, the predictions were somewhat better among women. With the growing concern regarding fairness in AI, future work may need to examine other demographics more closely, especially when generalising these results to non-UK and Irish populations.

We focused on depression occurring at middle age, which is commonly defined as ending at age 60 or 65 years. Censoring participants once they reached 65 years of age would have biased our analysis; younger participants would have had a longer follow-up time in which depression could be diagnosed, causing age to artificially appear as a strong predictor. To overcome this, we could have narrowed the range of eligible ages at baseline, decreasing the cohort size. Alternatively, we could have considered a 5-year follow-up window, which would have decreased the number of participants with an outcome of depression by approximately half, and would have raised the question of how to handle participants who were diagnosed with depression before 65 years of age but over 5 years after baseline. As most (97.3%) diagnoses of depression in our data were recorded at 65 years of age or younger, defining no further restrictions seemed to be a reasonable compromise.

Notably, incidence of depression may occur at a younger or older age. We expect that risk factors for teenage depression and old-age depression, despite sharing some commonalities with the ones identified here, are likely quite different overall, and so predicting the onset of depression at these ages would require further work.

These limitations notwithstanding, the current findings extend the study of risk factors for depression. First, they show that middle-aged individuals with no history of mental health problems can be identified as at risk of depression well before the time of diagnosis; more specifically, a relatively small set of people who are at a much higher risk of depression than the rest of the population can be identified. Second, our findings validate known risk factors for depression by employing a data-driven approach. Third, they show the importance of basing such predictions on multiple features. Such multiple-feature models appear to be robust to changes in demographic parameters (place of birth and gender) and depression assessment method.

Our findings have clinical implications regarding the development of strategies for mitigating the risk of depression. The identified risk factors may represent multiple vulnerabilities independently increasing the risk of developing depression in middle age, and indicate targets for multicomponent prevention strategies. We suggest that a concise set of questions can form the basis for a routinely administered questionnaire for monitoring mental health.

There are two important implicit assumptions in this work that need to be investigated further before considering deployment of such methods. The first is that predicting future depression is beneficial to the clinical process. At the very least, any intervention based on such predictions should be done with caution and care, especially considering their current precision. Simply telling a person that they are at risk may be detrimental, increasing rather than reducing their risk of developing depression. Moreover, the cost-effectiveness of any investment in such an approach should be weighed against the alternatives. For example, it is possible that rather than investing in a large survey for identifying people at risk for future depression, it would be more effective to improve service linkage for people who have experienced life events that are likely to trigger depression, such as redundancy or bereavement.

The second assumption is that predictions based on machine-learning methodology are preferable to alternative methods for prediction. For example, it might be the case that they are no better, in terms of accuracy or cost, than predictions made by the patients’ physicians, or even by the patients themselves. Hence, even if there is benefit in identifying people who are at risk for future depression, it remains to be proven whether this should be done by machine learning.

## Data Availability

Predictive models (similar to the one described in the supplementary) are available upon request from D.A.-S. Data used are property of the UK Biobank.

## Supplementary material

For supplementary material accompanying this paper visit http://doi.org/10.1192/bjo.2023.62. click here to view supplementary material
