# Supplementary material for: Predicting future onset of depression among middle-aged adults with no psychiatric history
Source: BJPsych Open. 2023 May 23;9(3):e85. doi: 10.1192/bjo.2023.62 (PMC10228240; doi:10.1192/bjo.2023.62)

# Supplementary Methods and Materials

### Sampling and Model Selection

In a preliminary study, using cross-validation over the development set, we considered the issues of sampling and the type of classification model to use. We concluded that, although the fraction of positive examples was small, neither under-sampling nor over-sampling improved the predictive power of the classification model. Additionally, comparing logistic regression to xgboost classifiers and neural nets suggested that, in our setting, logistic regression worked just as well as these alternatives; further, it converges more quickly and is more readily interpretable. Thus, logistic regression was the chosen classification model for the following analysis. Additional details regarding the abovementioned preliminary study are presented in the following supplementary materials.

**Selection of U.K. Biobank Data Entries**

Each U.K. Biobank (UKBB) data entry (or column) is entitled with a field name; for example, ‘duration walking for pleasure’. Entries are organised in a hierarchy, so that each field has a path leading to it, with each step down the path representing a more specific area of the overall topic. For example, the path to the aforementioned field is ‘Assessment Centre’ – ‘Touchscreen’ – ‘Lifestyle and environment’ – ‘Physical activity’ – ‘Duration walking for pleasure’.

Entries also have a ‘value type’, such as continuous, categorical, or date. The field ‘duration walking for pleasure’ is categorical, as participants were offered seven different answer options. In contrast, the field ‘duration of walks’ is integer, as participants were asked to estimate the number of minutes they spent walking on a typical day.

Categorical values are typically encoded with consecutive numbers, commencing at ‘1’, as well as negative values for options such as ‘Do not know’ or ‘Prefer not to answer’.

The UKBB offers a very convenient interface for exploring the various types of available data; this can be accessed at: <https://biobank.ndph.ox.ac.uk/showcase/search.cgi>

Data entries were selected as potential features for this study if they met the following criteria:

1. The data were collected for at least 300,000 participants.
2. The data type was ‘categorical’, ‘integer’, or ‘continuous’.
3. If the data were categorical, there were, at most, 10 categories with positive values.
4. The path for the data field did not include the category ‘hospital inpatient’.

**Transformation of UK Biobank Data to Binary Features**

To model the participants and predict future onset of depression, we transformed the extracted UKBB data into binary feature vectors as follows. For columns for which the data were categorical, we encoded the answers as a 1-hot vector. Namely, for column *q* we defined a set of binary features *q_i_*, such that *q_i_*=1 if the participant selected the *i*th answer for *q* and at least 0*.*5% of the participants answered *i*. Otherwise, *q_i_*=0. In addition, we defined the binary feature *q_unanswered_*, which was ‘1’ if the participant did not provide a valid answer for *q* or selected an answer that less than 0*.*5% of the participants chose, and ‘0’ otherwise. We included this feature in the feature matrix if it represented the results of at least 0*.*5% of the participants.

For data with continuous values, we clustered these values into 10 bins, with each bin taking contiguous values and an approximately equal number of participants. The bin number to which a participant was assigned could be considered a categorical value, for which we applied the process described above. Finally, we added the age of the participant as a non-binary feature.

**Definition of Prediction Models**

Prediction models in this study differ in the way their features selected. Feature selection was performed in two steps: first, features with a very small correlation (<0*.*01) with the labels on the training data (indication of future depression) were removed. Then, using a chi-square test, the *k* best features were selected (for some selected value *k*). We denoted such models *Model_Best−k_* and, in general, added a numeric subscript denoting the number of selected features.

Additionally, we were interested in the predictive power of questionnaires beyond those that directly investigate mental health. We selected UKBB entries for which the title did not include the term ‘mental health’, and restricted the resultant model to features derived from these entries. This model was denoted *Model_NMH_*. For comparison, we also considered the predictive power of mental-health questions alone, and denoted the resultant model *Model_MH_*. Finally, exploring features that reflected lifestyle and environment, we trained a model on features derived from UKBB columns with the term ‘lifestyle’ in their path; we denoted this model *Model_LS_*.

**Evaluation Metrics**

Three measures of predictive success were considered in this work. First, the area under the receiver operator characteristic curve was used to compute the predictive success over the entire dataset. This curve plotted the predictions’ recall (the fraction of positive examples correctly identified) as a function of the false positive rate (the fraction of negative examples erroneously identified as positive).

However, of greater interest in this work was the identification of a small subset of people who were at risk of future depression; thus, it was also interesting to evaluate the predictive success over such a subset. A logistic regression model assigned each participant a probability of receiving a diagnosis of depression. We took the 5% of the participant sample with the highest probability and computed the precision over this subset (the fraction of this group who were indeed so diagnosed a year or more after the baseline assessment), and recall (the fraction of the overall sample who received such a diagnosis and were included in the high-risk subset).

More formally, the 5% of participants who, according to the model, were most likely to be diagnosed with depression at *least one year* after the baseline assessment were denoted as *M*_0_*_._*_05_. We were interested in the precision and recall for this set, which we denoted as ‘Pr-5%’ and ‘Re-5%’, respectively. We then denoted the set of all participants who were diagnosed with depression as *D*, and the intersection of *M*_0_*_._*_05_ and *D* as *T*_0_*_._*_05_*,* that is those who were correctly predicted to be at risk. Then, Pr-5% is the ratio between the sizes of *T*_0_*_._*_05_ and *M*_0_*_._*_05_, while Re-5% is the ratio between the sizes of *T*_0_*_._*_05_ and *D*.

**Explicit Prediction Model**

To build an explicit prediction model, we used a slightly different methodology than that described above. We were interested in selecting a concise set of UKBB data entries for the model, rather than a concise set of features (recall that each data entry defines multiple features). Hence, we proceeded as follows:

Initially, the data entry that independently gave the best Pr-5% score (as per Table 2) was selected. Then, more data entries were added using nine steps of forward search (only entries featuring examples from the best 100 features were considered). That is, at each step we examined all data entries that were not yet selected and, for each available entry, we computed the precision of the model when the features associated with this data entry were added to the features allocated thus far. We then selected the data entry that yielded the highest AUC score and added it. We continued in this way until 10 different data entries were collected.

The data set was split into three – ‘train’ (70%), ‘dev’ (15%), and ‘test’ (15%). Models were trained on the train set, and data entries were selected based on the AUC over the dev set. After collecting 10 data entries, the resultant model was evaluated on the test set, yielding an AUC of 0.787, a Pr-5% of 0.118, and a Re-5% of 0.27.

Supplementary Table 1 lists the 10 data entries, and the weights assigned to each of the associated features, in the logistic regression model. Higher scores indicate higher risk of future depression.

It is important to note that this is **a** model for predicting depression, rather than **the** model for predicting depression. As a result of high correlations among features, similarly successful models can be constructed using different features and weights.

**Supplementary Table 1.**

Description of a logistic regression model for predicting future depression. UKBB fields were selected greedily using a forward-search.

| **UKBB field** | **UKBB column** | **Response** | **Weight** |
| --- | --- | --- | --- |
| Seen doctor (GP) for nerves, anxiety, tension or depression | 1173 | No | −0.52642 |
| Seen doctor (GP) for nerves, anxiety, tension or depression | 1173 | Yes | 0.606072 |
| Seen doctor (GP) for nerves, anxiety, tension or depression | 1173 | Do not know/unanswered | −0.08145 |
| Frequency of depressed mood in last 2 weeks | 1157 | Not at all | −0.40193 |
| Frequency of depressed mood in last 2 weeks | 1157 | Several days | 0.005014 |
| Frequency of depressed mood in last 2 weeks | 1157 | More than half the days | 0.084832 |
| Frequency of depressed mood in last 2 weeks | 1157 | Nearly every day | 0.396805 |
| Frequency of depressed mood in last 2 weeks | 1157 | Do not know/unanswered | −0.08652 |
| Number of self-reported non-cancer illnesses | 432 | 0 | −0.35685 |
| Number of self-reported non-cancer illnesses | 432 | 1 | −0.29602 |
| Number of self-reported non-cancer illnesses | 432 | 2 | −0.26028 |
| Number of self-reported non-cancer illnesses | 432 | 3 | −0.25546 |
| Number of self-reported non-cancer illnesses | 432 | 4+ | -0.066 |
| Overall health rating | 1201 | Excellent | -0.42968 |
| Overall health rating | 1201 | Good | -0.17313 |
| Overall health rating | 1201 | Fair | 0.05042 |
| Overall health rating | 1201 | Poor | 0.315137 |
| Overall health rating | 1201 | Do not know/unanswered | 0.235452 |
| Own or rent accommodation lived in | 701 | Own outright | −0.27628 |
| Own or rent accommodation lived in | 701 | Own with a mortgage | −0.02759 |
| Own or rent accommodation lived in | 701 | Rent - from local authority | 0.318122 |
| Own or rent accommodation lived in | 701 | Rent - from private landlord | 0.066676 |
| Own or rent accommodation lived in | 701 | rent free | 0.013769 |
| Own or rent accommodation lived in | 701 | Other/unanswered | −0.09649 |
| Fed-up feelings | 1121 | No | −0.22047 |
| Fed-up feelings | 1121 | Yes | 0.172594 |
| Fed-up feelings | 1121 | Do not know/unanswered | 0.046079 |
| Getting up in morning | 860 | Not at all easy | 0.074291 |
| Getting up in morning | 860 | Not very easy | −0.08172 |
| Getting up in morning | 860 | Fairly easy | −0.16719 |
| Getting up in morning | 860 | Very easy | −0.15445 |
| Getting up in morning | 860 | Do not know/unanswered | 0.327274 |
| Seen a psychiatrist for nerves, anxiety, tension or depression | 1177 | No | −0.16873 |
| Seen a psychiatrist for nerves, anxiety, tension or depression | 1177 | Yes | 0.117203 |
| Seen a psychiatrist for nerves, anxiety, tension or depression | 1177 | Do not know/unanswered | 0.049731 |
| Taking other prescription medications | 1303 | No | −0.05967 |
| Taking other prescription medications | 1303 | Yes | 0.338394 |
| Taking other prescription medications | 1303 | Do not know/unanswered | −0.28053 |
| Sleeplessness / insomnia | 872 | Never/rarely | 0.126929 |
| Sleeplessness / insomnia | 872 | Sometimes | 0.290061 |
| Sleeplessness / insomnia | 872 | Usually | 0.415266 |

GP: general practitioner; UKBB: UK Biobank

Supplementary Fig 1. Evaluation statistics as a function of the number of features selected for a logistic regression model (averaged over 10 folds of cross-validation with an 70/30 random split).

1. AUC: area under RoC curve; (b) Pr-5%: precision at 5%; (c) Re-5%: recall at 5%; RoC: receiver operating characteristic


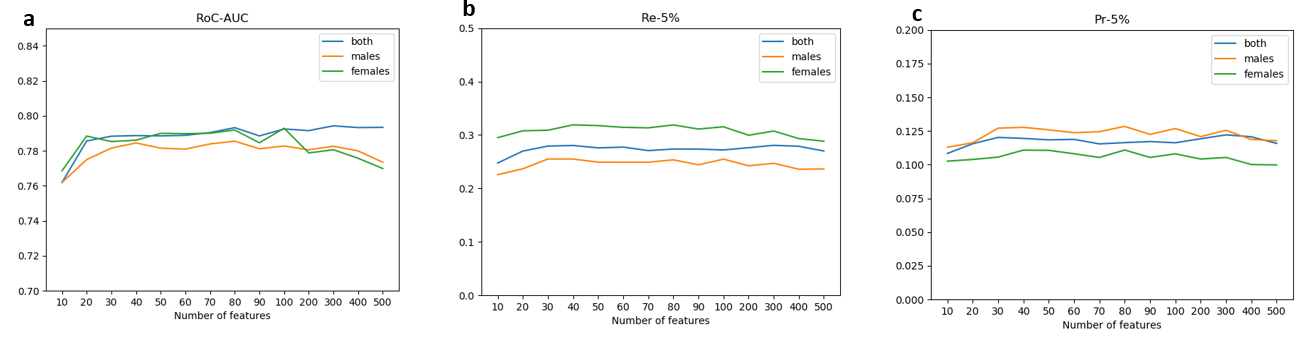

Supplement: Supplementary file 1 [file S2056472423000625sup001.docx]
